# In vivo mitral valve repair for the transplanted donor heart in orthotopic heart transplantation

**DOI:** 10.1186/s13019-024-02788-7

**Published:** 2024-05-13

**Authors:** Kazuma Handa, Yusuke Misumi, Daisuke Yoshioka, Shunsuke Saito, Masashi Kawamura, Takuji Kawamura, Ai Kawamura, Takashi Yamauchi, Shigeru Miyagawa

**Affiliations:** Department of Cardiovascular Surgery, Osaka University, Graduate School of Medicine, Yamada-Oka 2-2, Suita, Osaka Japan

**Keywords:** Orthotopic heart transplantation, Mitral valve regurgitation, Concomitant mitral valve repair

## Abstract

A 53-year-old woman with the dilated phase of hypertrophic cardiomyopathy underwent orthotopic heart transplantation. The donor heart was evaluated as normal preoperatively without mitral regurgitation or the left atrium dilation, transplanted using the modified bicaval technique. Although the heart beat satisfactorily after aortic declamping, massive mitral regurgitation was observed without any prolapse or annular dilation. Because of the difficulty in weaning from cardiopulmonary bypass, a second aortic cross-clamp was applied, and we detached the inferior vena cava and the right side of the left atrial anastomosis to approach the mitral valve, obtaining a satisfactory exposure. No abnormalities were observed in the mitral valve leaflets, annulus or subvalvular apparatus. Subsequent in vivo mitral annuloplasty using prosthetic full ring successfully controlled the regurgitation, and the patient was easily weaned from cardiopulmonary bypass. She discharged to home with good mitral valve and cardiac functions. And the patient has been doing well without any recurrence of MR or heart failure for over a year after surgery.

## Introduction

Bench mitral valve repair before heart transplantation for preoperatively identified mitral regurgitation (MR) has been reported [1–9]. However, the occurrence of severe MR following aortic declamping is extremely rare in a normal donor heart. We present a case in which massive MR occurred after declamping in a transplanted donor heart that had been evaluated as normal preoperatively, and mitral annuloplasty (MAP) was performed to control the MR under a second cross-clamp.

## Case report

A 53-year-old woman with the dilated phase of hypertrophic cardiomyopathy underwent orthotopic heart transplantation 6 years after implantation of a HeartMate II (HMII) (Thoratec Corp., Pleasanton, California) left ventricular (LV) assist device. The condition of the donor heart was evaluated as normal preoperatively by transthoracic echocardiography: LV asynergy was not observed, the LV diastolic/systolic diameter was 41/27 mm, the ejection fraction was 64%, aortic regurgitation was trivial, MR was trivial, the mitral valve structure was normal, and the left atrial diameter was also normal. The donor was matched for weight and the left atrium diameter did not show any significant difference between the donor and the recipient. Preoperative computed tomography of the donor heart revealed no calcified lesions in the coronary arteries. Bench analysis of the mitral valve showed no abnormality. During procurement of the donor heart, a novel cold (4 °C) cardioplegic solution (Celsior) was used for arrest and storage. There were no problems with infusion administration or transport. No continuous perfusion device was used in this case.

**Fig. 1:**
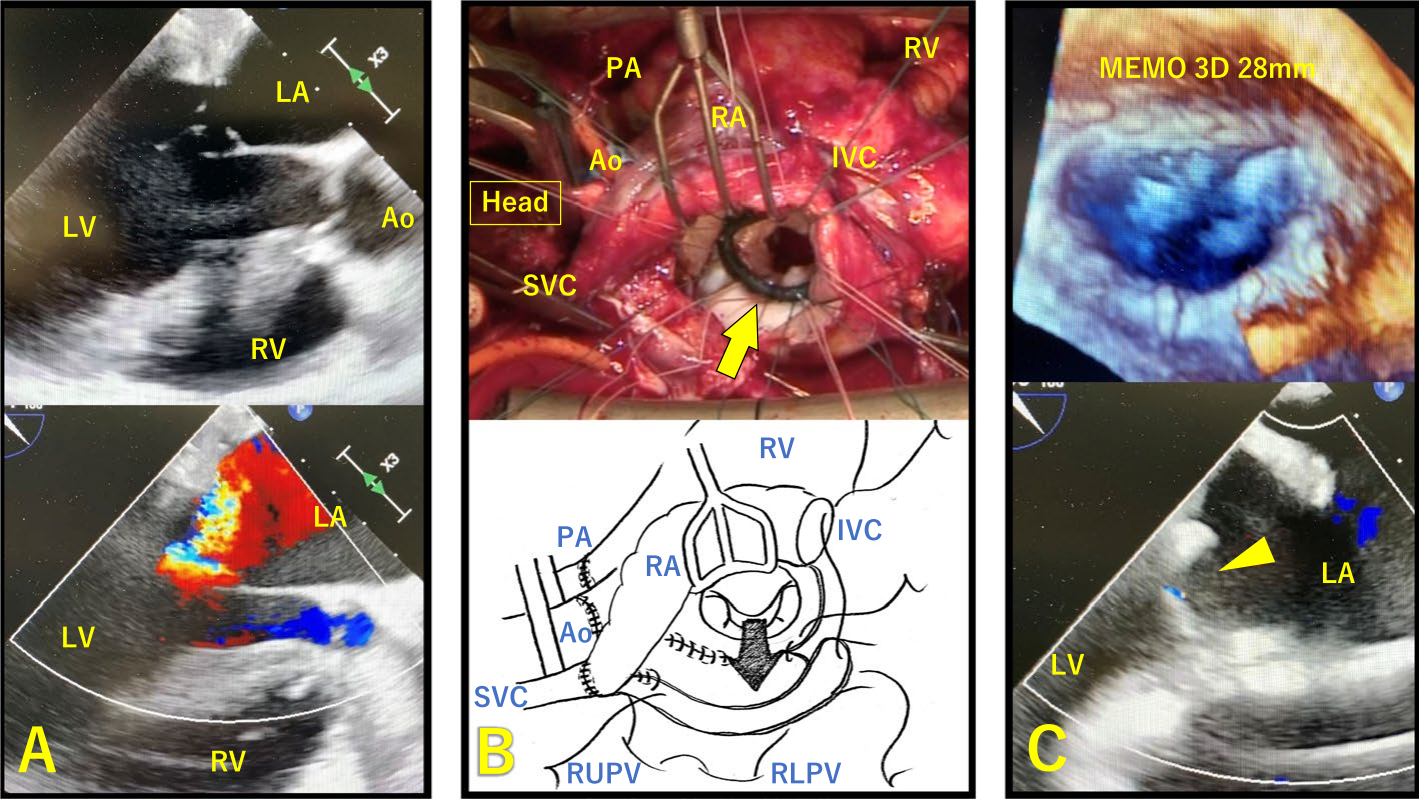
**A** Intraoperative TEE revealed no prolapse but showed severe MR originating from the center with mild tethering at the anterior and posterior leaflets, resulting in a shallow coaptation length. **B** We detached the IVC anastomosis and partially dissected the right side of the left atrial anastomosis to approach the mitral valve (arrow), obtaining satisfactory exposure. **C** Mitral annuloplasty was performed using one downsized 28-mm Memo 3D ring (Sorin Biomedica Cardio S.r.L., Saluggia, Italy), and TEE confirmed that the MR was well controlled (arrowhead). *TEE, transesophageal echocardiography; MR, mitral regurgitation; Ao, aorta; PA, pulmonary artery; LA, left atrium; LV, left ventricle; RA, right atrium; RV, right ventricle; SVC, superior vena cava; IVC, inferior vena cava; RUPV, right upper pulmonary vein; RLPV, right lower pulmonary vein*

The donor heart was transplanted using the modified bicaval technique. Although the heart beat spontaneously and satisfactorily after aortic declamping (total cold ischemic and preservation time: 212 min), massive MR was observed. Transesophageal echocardiography revealed no prolapse but showed severe MR originating from the center with mild tethering of the anterior and posterior leaflets, resulting in a shallow coaptation length (Fig. 1A). There was no other cause of MR, such as right ventricular pacing, LV asynergy or dyssynchrony in the transplanted heart, or abnormal QRS morphology on the electrocardiogram. Based on this information, the most probable cause of functional MR appears to be myocardial stunning resulting from prolonged cold ischemia. We monitored the MR under cardiopulmonary bypass for a period, expecting a reduction; however, no improvement was noted. Because the severe MR made weaning from cardiopulmonary bypass difficult, surgical intervention on the mitral valve was considered necessary. A second aortic cross-clamp was applied, and antegrade cold (15 °C) blood cardioplegic solution was administered to achieve cardiac arrest, and then intermittent retrograde cold (15 °C) blood cardioplegic solution was administered every 20 min for maintenance of cardiac arrest.

Considering the limited visualization of the mitral valve in a donor heart with a normal left atrial diameter and the aim of preserving potential future access sites by avoiding atrial incision, we did not utilize the right-side left atrial approach or the transseptal approach. Instead, we detached the inferior vena cava anastomosis and dissected approximately two-thirds of the right side of the left atrial anastomosis to approach the mitral valve, obtaining satisfactory exposure (Fig. 1B). No abnormalities were observed in the mitral valve leaflets or subvalvular apparatus, and a sufficient distance was maintained between the left atrial anastomosis and the mitral annulus. No distortion of the geometry of the mitral annulus caused by the left atrial anastomosis was observed, and there was no evidence of annular dilation. During the saline test, regurgitation was observed from the center with tethering of the anterior and posterior leaflets, diagnosed as the functional MR. and subsequent MAP was performed using one downsized 28-mm Memo 3D ring (Sorin Biomedica Cardio S.r.L., Saluggia, Italy) (Fig. 1C). After declamping (cardioplegic arrest time: 78 min), transesophageal echocardiography showed that the MR was successfully controlled. The patient was easily weaned from cardiopulmonary bypass.

The peak creatine kinase concentration was 553 U/L, and the creatine kinase MB isoenzyme concentration was 24.5 U/L. Postoperative transthoracic echocardiography revealed the following: LV diastolic/systolic diameter, 33/21 mm; ejection fraction, 68%, no LV asynergy; no MR or stenosis; and a mean transmitral pressure gradient of 2.5 mmHg, indicating good cardiac function, and discharge to home. The patient has been doing well without any recurrence of MR, exhibiting good cardiac function, and showing no signs of infection for over a year after surgery.

The patient provided written informed consent for publication of this case report. The Osaka University Hospital Clinical Research Ethics Committee approved the case report and publication of data (approval number: 16105; approval date: November 2, 2016).

## Discussion

Several reports have described the performance of bench mitral repair when MR was identified in the donor heart prior to transplantation [1–9]. Prieto et al. [5] described bench repair on donor hearts without significant preoperative MR but with mitral annular dilation. However, the occurrence of severe MR following aortic declamping has been rarely reported in a normal donor heart. In this case, the preoperative cardiac function of the donor heart was evaluated as normal with trivial MR, and there was no evidence suggestive of coronary artery disease or significant myocardial injury. Additionally, there was no abnormality of the mitral valve structure, including the annulus and subvalvular apparatus.

Functional severe MR can reportedly occur after declamping during open heart surgeries other than mitral valve procedures, such as coronary artery bypass grafting and aortic valve replacement, even when cardiac function is normal [10]. One possible cause is dyssynchrony due to right ventricular pacing. In some cases, however, reestablishment of cardiopulmonary bypass and additional concomitant mitral valve surgery are necessary when severe MR remains. This suggests that impaired myocardial function following cardiopulmonary bypass might contribute to diastolic dysfunction. In the current case, although the postoperative CK-MB level was within the normal range, indicating no myocardial necrosis, the extended cold ischemia time of 3.5 h suggested that myocardial stunning due to prolonged ischemia was the most likely cause of functional mitral regurgitation. The widespread adoption of continuous perfusion systems could help maintain myocardial protection even during anticipated prolonged ischemic times, potentially preventing cases such as the present case. And other possible reason for the lack of detection of MR in the preoperative evaluation is the decrease in LV afterload due to decreased sympathetic activity after brain death, resulting in underestimation of the MR.

Conventional approaches such as transseptal or right-sided left atrial approaches are commonly used for mitral valve surgery during the post-transplant period [11, 12]. However, in this case, the approach was achieved by detaching the inferior vena cava anastomosis and partially left atrial suturing. This approach allowed for rapid and good visualization by simply cutting the sutures, and it provided the advantage of being able to promptly address any interference between the left atrial suture line and the mitral valve. Despite the need for re-anastomosis, this approach was considered useful. The benefits of a right-sided left atrial incision or a transseptal approach are that the sutures do not need to be dissected, and surgeons are already familiar with the usual method. However, a disadvantage is that if there is distortion of the geometry of the mitral annulus caused by left atrial anastomosis, it cannot be released. The current approach, which involves dissecting the IVC anastomosis, has the advantage of providing a clear view, even in cases where the left atrium is not enlarged, as in this case. This approach also allows for simultaneous repair of the distortion of the mitral annulus caused by atrial anastomosis. Additionally, the right-sided left atrial incision or transseptal approach can be preserved for future use. On the other hand, disadvantages include the large number of suture points and unfamiliar approach method in routine surgery.

While heart transplantation is an established treatment for severe heart failure, the shortage of donor hearts is a major concern owing to the increasing number of patients with heart failure. One solution is to broaden the criteria for accepting donor hearts [13, 14]. Bench valve repair [1–9] may be a preferable option when simple valvular disease is identified preoperatively, as reported in the literature. However, this often requires the use of prosthetic valves or rings, which should be minimized due to the high risk of infection from post-implantation immunosuppressive drugs. In cases where preoperative valvular disease is not obvious, as in our case, it may be beneficial to proceed with transplantation and assessment of valvular disease after declamping. This simultaneous intervention for valvular disease in the donor heart could potentially expand the donor eligibility criteria and alleviate donor shortages.

Even if the preoperative donor heart evaluation is normal and MR is not observed, attention should be paid to the occurrence of functional MR after declamping, if the cold ischemic time is prolonged. Although spontaneous improvement is possible, MAP may be a good method of MR control. In addition, it may be possible to secure a good view of the mitral valve in the normal-diameter left atrium by removing the IVC and part of the left atrial anastomosis.

## Conclusion

Even when there is no evidence of preoperative MR in the donor heart, severe MR can occur after reperfusion, especially if ischemia time is prolonged, and careful attention is needed. In this case, MR was successfully controlled by MAP with a full prosthetic ring.

## Data Availability

The authors declare that all data in this article are available within the article.

## Abbreviations

MR: Mitral regurgitation
MAP: Mitral annuloplasty
LV: Left ventricle
